# Signal Transduction Inhibitors as Promising Anticancer Agents

**DOI:** 10.1155/2015/584170

**Published:** 2015-05-21

**Authors:** Raj Kumar, Cedric Dos Santos, Tarunveer Singh Ahluwalia, Sandeep Singh

**Affiliations:** ^1^Laboratory for Drug Design and Synthesis, Centre for Chemical and Pharmaceutical Sciences, Central University of Punjab, Bathinda Punjab 151001, India; ^2^Hematology-Oncology, Perelman School of Medicine, University of Pennsylvania, 720 BRB-II/III, 421 Curie Boulevard Philadelphia, PA 19104-6160, USA; ^3^The Novo Nordisk Foundation Centre for Basic Metabolic Research, Section of Metabolic Genetics, Faculty of Medical and Health Sciences, University of Copenhagen, Universitetsparken 1, DIKU Building, 2100 Copenhagen, Denmark; ^4^Centre for Genetic Diseases and Molecular Medicine, Central University of Punjab, Bathinda Punjab 151001, India

Cancer is a group of diseases sharing common features like unrestrictive growth, metastasis, and angiogenesis; however the basic signal transduction pathways are deregulated to such an extent that every cancer case itself poses new challenges for the therapeutics. Worldwide approximately 7.6 million people died of cancer in year 2008 and it has been projected that 13.1 million deaths will be due to cancer by year 2030.

Understanding the disease etiology and dysregulation of tissue microenvironment, signal transduction pathways are the potential directions, which may help us find the possible cure for the disease. However, recent advances in cancer therapeutics are proving to be beneficial for the patients but there is still a lot to be desired. Continuous research worldwide is focusing on developing better therapeutics as well as finding novel druggable targets for better efficacy. Another recent development is novel multitarget drugs, which may increase the efficacy manyfold.

In the current special issue a total of twelve articles were received which were of the highest quality indicating the level of interest worldwide on the issue. The articles were having potential topics including (i) understanding of signal transduction alteration in cancer development, (ii) experimental studies highlighting the role of various cell signaling molecules involved in carcinogenesis, (iii) mechanistic studies involving better (novel) animal/cell culture models for signal transduction studies in cancer, and (iv) evaluation of synthetic and natural products as cell signaling inhibitors in cancer development, angiogenesis, and metastasis. Out of the twelve articles received, five were accepted for publication in the special issue.

In study entitled “Deguelin Induces Apoptosis by Targeting Both EGFR-Akt and IGF1R-Akt Pathways in Head and Neck Squamous Cell Cancer Cell Lines,” Y. Baba et al. investigated potential anticancer mechanisms of a retinoid compound named deguelin derived from the African plant* Mundulea sericea* (Leguminosae) in head and neck squamous cell carcinoma (HNSCC). The flow cytometry data showed accumulation of proapoptotic cells in deguelin treated cells. The compound inhibited IGF-1 and EGF induced Akt activation. Cell death induced by the compound was reported to be via reduction of phospho-IGF1R, Akt, and ERK1/2. Overall, the study showed potential mechanisms behind antitumor activity of deguelin and suggested that it may be applicable therapeutic strategy for head and neck squamous cell cancer.

S. J. Assinder et al. proposed novel role of negative regulators of receptor tyrosine kinase in clinical settings. The study “Cosuppression of Sprouty and Sprouty-Related Negative Regulators of FGF Signalling in Prostate Cancer: A Working Hypothesis” targeted FGF RTK signaling which is commonly involved in prostate cancer. The authors explored potential role of sprouty and sprouty-related antagonists in prostate intraepithelial neoplasia using various knock-out mice models. By performing various* in vivo* and clinical analyses, the authors conclude that in prostate cancer sprouty and sprouty-related antagonists are significantly repressed demonstrating the importance of negative regulators of RTK and highlighted their importance for future pharmacopeia.

In research article “Biological and Molecular Effects of Small Molecule Kinase Inhibitors on Low-Passage Human Colorectal Cancer Cell Lines,” F. Lange et al. tested various small molecule kinase inhibitors such as vemurafenib, trametinib, perifosine, and regorafenib in 4 cancer cell lines (CRC) established from colon cancer patients. The mutant BRAF inhibitor vemurafenib and MEK1/2 inhibitor trametinib efficiently inhibited DNA synthesis in BRAF mutant cells. On the other hand, the AKT inhibitor perifosine was effective in three cell lines but the fourth cell line was resistant to it. Regorafenib, which is multikinase inhibitor, suppressed proliferation in all the cell lines irrespective of KRAS, BRAF, PIK3CA, and TP53 mutations or expression. In conclusion, the authors stressed on use of low-passage CRC cell lines for preclinical investigations to test small molecule inhibitors.

In the paper “Roles of ER*β* and GPR30 in Proliferative Response of Human Bladder Cancer Cell to Estrogen,” W. Huang et al. probed potential involvement of estrogen receptors in progression of bladder cancer. Cells were treated with different doses of 17*β*-estradiol followed by cell proliferation analysis. Further mechanistic insights indicated that 17*β*-estradiol effect is EGFR-MAPK pathway independent and primarily happens due to GPR30. On the other hand, c-FOS, BCL-2, and cyclin D1 expression was increased by estradiol treatment that was independently associated with EGFR-MAPK pathway.

M. Xie et al. in their study entitled “Progesterone and Src Family Inhibitor PP1 Synergistically Inhibit Cell Migration and Invasion of Human Basal Phenotype Breast Cancer Cells” demonstrated antimetastatic effects of PP1 in aggressive breast cancer cell lines. The authors detected moderate expression levels in brain-metastatic BPBC cell line MB231Br, which was derived from the parent mPR*α* undetectable MB231 cells. The work provided interesting and novel findings towards development of novel anticancer agents targeting nuclear hormonal receptors and endocrine-resistant breast cancers.

*Raj Kumar* *Cedric Dos Santos* *Tarunveer Singh Ahluwalia* *Sandeep Singh*

